# Progress in the Analysis of Food Allergens through Molecular Biology Approaches

**DOI:** 10.3390/cells8091073

**Published:** 2019-09-12

**Authors:** Mariateresa Volpicella, Claudia Leoni, Maria C.G. Dileo, Luigi R. Ceci

**Affiliations:** 1 Department of Biosciences, Biotechnologies and Biopharmaceutics, University of Bari, Via Amendola 165/a, 70126 Bari, Italy; c.leoni@ibiom.cnr.it; 2 Institute of Bioenergetics, Biomembranes and Molecular Biotechnologies, Italian National Research Council, Via Amendola 165/a, 70126 Bari, Italy; 3 Department of Biology, University of Bari, Via Amendola 165/a, 70126 Bari, Italy; mariacristina.g.1993@gmail.com

**Keywords:** food allergen, food allergy, epitope mapping, component resolved diagnosis, immunotherapy

## Abstract

Food allergies associated with class E immunoglobulins (IgE) are a serious health problem that affects between 1% and 10% of the population of developing countries, with a variability that depends on the geographical area and age range considered. These allergies are caused by a cross-link reaction between a specific food protein (the allergen) and the host IgE. Allergic reactions can range from mild itching to anaphylactic shock and there are no clues to predict the effects of an allergen. Strict avoidance of allergenic food is the only way to avoid possible serious allergic reactions. In the last 30 years a growing number of molecular studies have been conducted to obtain information on the diffusion of food allergens and to establish the structural basis of their allergenicity. At the same time, these studies have also allowed the development of molecular tools (mainly based on synthetic peptides and recombinant allergens) that can be of great help for diagnostic and therapeutic approaches of food allergies. Accordingly, this review focuses on advances in the study of food allergens made possible by molecular technologies and how results and technologies can be integrated for the development of a systematic food molecular allergology. The review may be of interest both to scientists approaching this field of investigation and to physicians who wish to have an update on the progress of research in diagnosis and therapy of food allergies.

## 1. Introduction

Significant progress has been made in molecular allergology since the first allergen coding sequence was cloned in the late 1980s [1]. Nowadays, molecular biology technologies, like DNA cloning, protein expression and structural analysis, microarray analysis, peptide synthesis, directed evolution and next generation sequencing, just to mention the main ones, offer a series of effective resources to study the molecular and structural basis of allergies and provide cutting-edge tools for their diagnosis and therapy.

In this scenario, food allergies offer an interesting field of research and application. In fact, in addition to their wide diffusion and possible severity, food allergens have, in some cases, a very compact and heat resistant structure, able to resist to the food cooking process.

**Figure 1:**
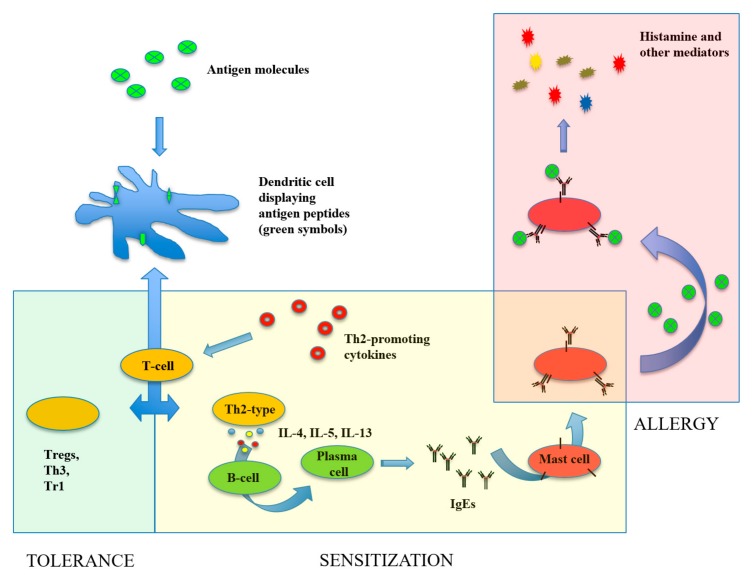
The onset of food allergy reactions. In the gastrointestinal tract, the food antigenic proteins are captured by dendritic cells (DCs) and hydrolysed into small peptides, which are displayed on the cell surface associated with the molecules of the major histocompatibility complex. After migration to the mesenteric lymph nodes, DCs interact with T cells to initiate the adaptive immune response. In the absence of particular stimulatory signals, T cells differentiate into suppressive T cells (Treg, Th3, Tr1) originating a tolerance status (green area). It may happen, however, that in the presence of Th2-promoting cytokines (occurring in the gastrointestinal tract, in the skin and respiratory tract, especially in the presence of epithelial injuries) T cells become Th2-type helper T cells. The Th2-type cells migrate to different tissues and produce specific cytokines (IL-4, IL-5, IL-13) that can induce differentiation of food antigen-specific B cells into plasma cells producing antigen-specific IgE. In the so-called sensitization phase (yellow area), the produced IgE molecules bind to specific receptors on the tissue mast cell. From now on, a subsequent interaction of the antigen with its specific IgE bound to the mast cells will cause mast cell degranulation with release of molecules, such as histamine, prostaglandins, tryptase and cytokines, leading to allergic reaction (red area). More details on the pathogenesis of food allergy can be found in specific review articles [2,3].

In general, food allergies comprise both IgE- and non-IgE-mediated immune disorders, occurring after exposition to the allergenic food. While non-IgE-mediated food allergies correspond to pathologic conditions of specific tissues mainly involving food antigen-specific T-cell responses (e.g., celiac diseases), IgE-mediated allergies have different manifestations, including mild pruritus, gastrointestinal symptoms and also life-threatening systemic anaphylaxis reactions. Figure 1 shows a schematic representation of the cellular and molecular processes underlying the onset of IgE-mediated food allergic reactions.

The presence of antigen specific IgE in sera of allergic patients is generally the effect of a first allergic sensitization occurred by exposition to oral (food) or aero (pollen) allergens. Subsequent exposure to homologous food allergens can cause the onset of allergic reactions [2,3,4,5]. This review will only deal with IgE-mediated food allergies.

Currently, hundreds of food allergens have been identified (739 according to the Allergen Online database, http://www.allergenonline.org/ [6], updated on 10 February, 2019) and “food allergens” have been the subject of many review articles. In addition to general reviews about “food allergy” [2,4,5,7,8] or classification and characterization of single families or groups of food allergens [9,10,11,12,13,14,15], specific reviews deal with recombinant allergens and their use in component resolved diagnosis (CRD) [16,17,18,19,20,21,22], immunotherapy [23,24,25,26,27,28] or both [29,30,31].

**Figure 2:**
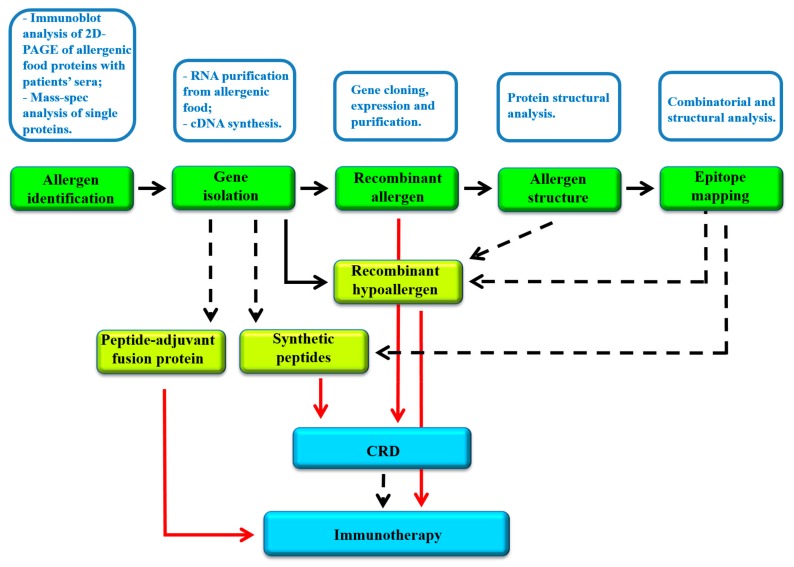
Scheme of a possible experimental workflow of molecular approaches for the identification of allergens from food sources, their characterization and development of diagnostic and therapeutic tools. Main procedures are reported in white boxes above the corresponding product, indicated in the green boxes. The results obtained can be then used for the development of molecular tools (yellow boxes) required for CRD and immunotherapy. Dashed arrows indicate the use of data. Red arrows indicate the application of the molecule to CRD or immunotherapy.

Recombinant allergens play an important role in modern allergology. In fact, besides being the subject of studies aimed at establishing possible structure/allergenicity relationships, they are also useful tools for diagnostic and therapeutic applications. Figure 2 shows the scheme of a possible workflow in which different molecular approaches are integrated to: (i) identify and characterize the allergens of a specific allergenic food and (ii) develop possible diagnostic and therapeutic procedures. The following paragraphs provide an overview of the several studies conducted with molecular biology approaches in food allergology. In particular, the experimental procedures that can be performed once the sequence of an allergen (as a protein or as a gene) is acquired will be taken into consideration. The results obtained, such as allergen cloning, structural characterization and epitope mapping, are particularly valuable for application in diagnostic (CRD) and therapeutic procedures (immunotherapy).

## 2. Epitope Mapping by Molecular Approaches

Numerous studies addressed the identification of epitopes (i.e., regions of the allergen recognized by specific antibodies) in allergenic molecules. Identification and characterization of allergen epitopes is a key step for understanding the dynamics of the allergenic immune response and identifying biomarkers or therapeutic targets for specific immunotherapy [32]. Epitopes are generally divided in linear or conformational, depending on whether they correspond to a continuous amino acid sequence or to a group of amino acids variously distributed in the primary structure of the allergen and which are oriented in close proximity within each other in the three-dimensional structure of the folded allergen [33].

Linear epitopes can be identified by immuno-screening assays of allergen-derived overlapping synthetic peptides with the sera of allergic patients [34]. One of the first studies using this approach allowed to identify an epitope of the major allergen Der p II from the house dust mite *Dermatophagoides pteronyssinus* [35]. It was carried out by using 14 synthetic peptides, 15 amino acids in length, overlapping by five residues and spanning the entire sequence of the allergen. The peptides were coupled to CNBr-activated Sepharose-4B and tested using human IgE antisera. The IgE-peptide interaction occurred mainly with the peptide comprising residues 65–78. Several companies are available today for the production of custom peptide arrays.

The identification of conformational epitopes is more demanding. It is based on different approaches, including molecular analysis by combinatorial techniques (see below) and structural and bioinformatic analysis of the allergen molecules. These approaches are of mutual benefit and are often used in combination.

**Figure 3:**
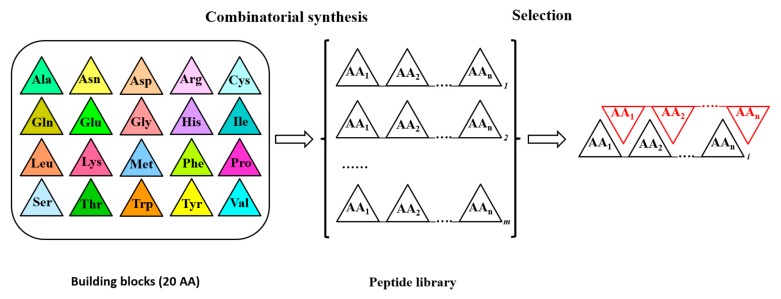
Synthesis and screening of a combinatorial peptide library. The synthesis of an n-mer peptide combinatorial library is schematized. Coloured triangles stand for specific amino acids (AA) used for a randomized synthesis of n-mer peptides. White triangles represent any possible amino acid in synthesized peptides. n = peptide length; m = total number of peptides in the library (m = 20^n^). Amino acids in red triangles constitute a specific peptide used as a bait for the selection from the library of the peptide with the highest affinity (the length of the bait is not necessarily “n” amino acids). In general, any kind of molecule able to interact with peptides can be used as a bait. The library or the bait are fixed on a solid support to allow selection. In the first case the positions of each peptide on the solid support is known. In DNA based combinatorial libraries, a selected number of codons is randomized in order to obtain after expression the corresponding combinatorial peptide library. In phage display libraries, for example, the gene of interest (containing the chosen degenerated codons) is fused with the gene for a phage surface protein. In this way, after expression of phage particles in *Escherichia coli* cells, a combinatorial phage library is produced and can be used in the selection step.

In mutational analysis of proteins, combinatorial approaches allow the identification of amino acids critical for increasing (or modifying) a specific characteristic of the protein, such as activity, interactions with other molecules, etc. [36,37]. Combinatorial approaches adopt a series of molecular techniques (going from the direct peptide synthesis to more elaborated approaches such as error prone PCR, PCR with oligonucleotides carrying degenerated nucleotides in specific positions, DNA shuffling, phage display) capable of producing a large set of amino acid sequences for a specific region of the protein under analysis. The obtained mutant libraries are then used in directed evolution studies for the selection of mutants with the highest levels of the required property. Figure 3 schematizes the synthesis of a random peptide library and the selection of a single peptide by affinity capture with an immobilized peptide. Differently from the direct synthesis of a peptide library, the other combinatorial approaches mentioned above are used to obtain the gene of interest with mutated codons. While error prone PCR and gene shuffling yield novel gene sequences with a reduced control about the position of mutations, PCR with degenerated nucleotides and phage display allow to obtain novel gene sequences with mutated codons (up to complete randomization) in precise positions of the gene. In principle, in the phage display approach the gene of interest (or part of it) is randomized in a specific region and fused with the gene of a phage coat protein, resulting in the display of the randomized protein on the phage surface [37,38]. In addition, combinatorial libraries of random peptides fused to a coat protein of phages are also commercially available. In any case, this methodology allows the presentation of many different peptides on phage particles to be tested against allergen-specific antibodies.

In the specific field of food allergens, epitopes have been identified for several molecules using synthetic peptides and phage display approaches and review articles on the topic are already available [39,40]. An exemplificative case can be considered the study carried out on tropomyosin, a major crustacean allergen. In a first study, 46 overlapping peptides, 15 amino acids long and covering the whole tropomyosin of shrimp (*Penaeus aztecus*), were analysed by immunoreaction with the sera from 18 shrimp allergic subjects, allowing the identification of five major linear epitopes [41]. In a more recent article by Liu et al. [42] both the approaches (synthetic peptide and phage display screening) were used to identify epitopes in the tropomyosin molecule of mud crab (*Scylla serrata*). A group of 14 mimotopes (putative epitopes identified by phage display) were firstly identified by screening a commercial phage display library against a rabbit anti-*S. serrata* polyclonal antibody. By analysing the sequences of mimotopes and the sequence and the structure of tropomyosin, eight linear epitopes and seven conformational epitopes were deduced. Then, after exclusion of sequences containing trypsin cutting sites, twelve synthetic peptides were synthesised to test their allergenicity against the sera of ten crab allergic subjects. Among those, one linear epitope and two conformational epitopes were found to immuno-react with all the patients’ sera.

A complete list of epitopes identified in food allergens can be found at the IEDB database (Immune Epitope Database, www.iedb.org) [43]. IEDB is a complete resource of data on experimentally-determined epitopes, related to infectious diseases, allergy, autoimmunity and transplantation. The database contains data on B cell and T-cell epitopes retrieved by the analysis of the literature for human, nonhuman primate and rodent hosts, as well as a number of other animal species. Epitopes for food allergens represent about 53% of the B-cell and T-cell epitopes present in the database [44].

## 3. Epitope Prediction Analysis by Bioinformatic and Structural Tools

The prediction of epitopes is also based on advances in protein structural analysis and in bioinformatics. Although the two approaches can, in principle, provide independent tools, some predictive pipelines were developed that combine protein structural analysis with protein sequences analysis.

Identification of linear epitopes by sequence comparison between allergens can be carried out by common alignment programs, such as Clustal Omega [45]. It should be noted, however, that high sequence identity is only a prerequisite for the identification of possible linear epitopes, since different folding can change the effective exposition of epitopes in homologous proteins. Allergenicity of specific regions can also be evaluated by more sophisticated approaches. For example in IEDB [44] a predictive method based on evaluation of amino acid properties (such as hydrophilicity and chain flexibility) and the related propensity to form epitopes is available for the identification of novel linear epitopes in allergens.

Prediction of conformational epitopes is more difficult. Ideally, knowledge of protein 3D structures and epitopes in homologous allergens would allow an easy identification of possible epitopes in any new allergen. Unfortunately, the availability of these data is still relatively low and the results obtainable using existing prediction tools have low accuracy [40]. Clearly, data obtained by parallel epitope mapping assays can contribute to validate the prediction of conformational epitopes. Recently, Mishra et al. [46] using a combination of bioinformatic tools were able to predict three linear epitopes for an allergenic rice chitinase. To confirm their results, two synthetic peptides corresponding to the epitopes were tested for IgE binding capacity by ELISA assay using sera of allergic patients. Both the peptides were reactive, even if with different binding capacity, demonstrating the validity of the prediction.

An exhaustive list of bioinformatic tools for the prediction of linear and conformational epitopes can be found in the recent review by Liu and Sathe (2018) [40].

## 4. Recombinant Food Allergens Used for Component Resolved Diagnosis

Cloning and expression as recombinant molecules of the first allergens date back to the late 1980s. In a pioneering work Thomas et al. [1] cloned and expressed in *E. coli* cells the first gene for an allergen, the house dust mite allergen Der p 1. The successfully expressed recombinant protein was shown to react with a rabbit anti-Der p1 antiserum, suggesting the expression of a correctly folded IgE reactive protein. In the same period, the major allergen of white-face hornet venom (*Dolichovespula maculate*), Dol m V, was successfully cloned, expressed and purified [47]. Additionally, in this case, the proper folding of the recombinant molecule was demonstrated by immunoreactive assays, using mono- and polyclonal antibodies obtained from Dol m V immunized mice. Following, Valenta et al. [48] cloned and expressed in *E. coli* the genes for the birch-pollen allergen Bet v 1 and the birch profillin allergen Bet v 2. For these recombinant proteins, allergenicity was confirmed for the first time by immunoblots with sera of 100 patients allergic to birch pollen. Accordingly, the authors advanced the possibility to use recombinant allergens for in vitro allergy diagnostic tests, allowing a novel form of CRD. With the currently available cloning strategies, a recombinant allergen can be produced relatively easily and, once its allergenicity has been verified, it can be used for diagnostic purposes as it is, or even in mutated forms. To date, numerous allergens have been produced as recombinant molecules and are, in principle, available for CRD. Information for available recombinant food allergens can be obtained starting from the list of 739 food allergens reported in the Allergen Online database (see Introduction) that contains useful links to sequence databases.

In general, CRD allows the identification of allergen-specific IgE in patients’ sera using either a single molecule assay (simplex CRD) or an array of different molecules (multiplex CRD). The micro-chip-based array ImmunoCAP Solid phase Allergy Chip (ISAC) by Thermo Fisher Scientific offers the possibility to assay in a single experiment 112 different allergens. After the reaction, IgEs are quantified by a second reaction with fluorescence labelled anti-IgE antibodies. Quantification of IgEs is expressed semi-quantitatively in ISU-units, i.e., ISAC standardized units: < 0.3 ISU: very low IgE level; 0.3–0.9 ISU: low level; 1.0–14.9: moderate to high level; ≥ 15 ISU: very high level [20].

In the diagnosis of food allergies, CRD is particularly useful because it allows: (i) to detect the patient’s profile of sensitization; (ii) to quantify specific IgE (and, therefore, predict symptom severity), (iii) to verify the possibility of cross-reactions (IgE reactions with highly similar allergens from different sources), and (iv) to identify patients with low levels of specific IgE, who could be subjected to immunotherapy protocols.

CRD has proven especially useful in the diagnosis of peanut allergies since it can distinguish reactivities of the different allergens. Klemans et al. [49] demonstrated the diagnostic value of measuring IgE specific of the allergens Ara h 2 and Ara h 6 (two forms of conglutin) responsible for severe allergic reactions to peanut. Ara h 2 and Ara h 6 specific IgE were measured by ImmunoCAP ISAC. The diagnostic value of the specific IgE on population level was good for the two allergens. However, on individual level, 5% of the subjects showed contradicting results between the two tests, indicating the need to use both the test to avoid misdiagnosis.

Debates on the diagnostic accuracy of CRD technologies have also recently begun. In fact, even if high values of accuracy have been obtained for specific allergens, there is not a general standard of procedures and results in the available CRD studies. In their review Kim et al. [21] examined eleven studies in which CRD procedures were adopted for the diagnosis of allergies to different foods, such as cow’s milk, hen’s egg, peanut, hazelnut and shrimp. It resulted that only a limited number of components can be evaluated with high accuracy, in particular: cow’s milk α-lactoalbumin (Bos d 4), hen’s egg ovomucoid (Gal d 1), peanut Ara h 6, hazelnut 2s albumin (Cor a 14) and shrimp’s tropomyosin. Therefore, it emerged that the standardization of the CRD approaches (and their adoption as a routine test) still requires a larger number of studies conducted with homogeneous methodologies.

## 5. Polypeptides and Recombinant Food Allergens for Immunotherapy

It has been known since the early 1900s that allergy treatment can be pursued by the controlled administration of the eliciting allergen. Allergen-specific immunotherapy is based on repeated administrations to patients of increasing amounts of specific allergens (or part of them) that promote the synthesis of IgG antibodies, capable to compete with allergen-specific IgE. Additionally, in the case of immunotherapy, molecular techniques based on allergen-specific oligopeptides or single recombinant allergens allow standardization of the approach, overcoming the problems related to the use of allergens extracted from their natural sources. Allergen extracts do not guaranty consistent dosages, presence of all allergenic molecules (which may depend from the different extraction procedure), and the absence of unknown allergens (possible cause of new sensitizations) [28]. Immunotherapy for food allergies is still in an experimental status, but interesting results have already been obtained, using both peptides and recombinant molecules as immunizing agent [27].

Allergen-specific peptides for immunotherapy correspond to T cell epitopes. They can be selected from libraries of synthetic peptides by screening against patients’ T cell lines [50]. Peptide-based immunotherapy studies for egg ovoalbumin (Gal d 2) allergy were conducted in murine models using separately three T cell epitopes, or a mixture of them, administered by subcutaneous injection. In contrast to control, the mixture of Gal d 2 peptides was shown to be more effective in reducing the levels of histamine release in mice orally challenged with 20 mg of ovoalbumin [51].

T cell epitopes of the major peanut allergens Ara h 1 were also identified and used for establishing the efficiency of immunotherapy in murine models by intraperitoneal injections of variable doses of the peptides. Compared to controls, mice subjected to immunotherapy showed lower immunoreactions (lower histamine levels, milder allergic reactions and lower anaphylaxis cases) when challenged with peanut extracts (with 100 μg resulting as the most effective peptide dose) [52,53]. In the case of Ara h 2, a portion of the relative gene, encoding a peptide of 42 amino acids containing immunodominant B-cell epitopes and one T cell epitope, was fused with the gene for a surface S-layer protein of *Lactobacillus buchneri* acting as a carrier [54]. Despite the recombinant protein showed reactivity with Ara h 2-specific IgE from about 70% of the tested sera, it resulted ineffective in inducing the release of β-hexosaminidase, a marker of IgE-mediated cell degranulation, from sensitized rat basophil leukemia (RBL) cells at concentrations up to 100 ng/mL. For the same cells, Ara h 2 was able to induce release of β-hexosaminidase at only 10 pg/mL. The capacity of the fusion protein to induce the production of blocking IgG antibodies was also evaluated by ELISA inhibition assay using the sera of 20 peanut allergic patients. IgG antibodies (induced by immunization of rabbits with the fusion protein) inhibited only partially IgE-binding to the natural Ara h 2 and with different levels (more than 30% reduction observed for four sera, more than 20% for eight sera, and below 10% for eight sera) in comparison with the inhibition by anti-Ara h 2 rabbit IgG antibodies (48% ± 13.5% reduction). These results indicate that more than one allergen-peptide is probably needed to promote wider patient protection. For the cow’s milk allergen α(s1)-casein (Bos d 9) a mutational analysis was carried out to identify epitope-derived peptides with reduced IgE binding capacity and possibly effective in immunotherapy approaches. Peptides of 10–14 amino acids in length were synthesized with single or multiple amino acid substitutions and tested for their reactivity with sera from allergic patients. Although a general reduction in IgE binding capacity was obtained, it should be noted that, in some patients, a non-significant reduction of IgE binding was observed, indicating a heterogeneous pattern in IgE recognition [55]. Additionally, in the case of recombinant allergens, immunotherapy of food allergies has not yet been developed to the status of approved therapeutic protocols. This is mainly due to the unpredictable anaphylaxis reactions that can occur in individual patients.

In recent years, several hypo-allergenic allergens, obtained mainly by mutation of amino acids involved in IgE binding, have been produced to assess the possibility of their use in immunotherapy protocols. Successfully modified allergens have a reduced capacity to bind to IgE of patients’ sera when compared with wild-type allergens and, at the same time, maintain the ability to stimulate T cell proliferation. These are indispensable characteristics for the development of safe immunotherapy tools. Mutants of the peanut allergens Ara h 1, Ara h 2, Ara h 3, for example, were assayed for their capacity to bind peanut-specific IgE in immunoblotting assays with sera of peanut allergic patients. Compared to their wild-type counterpart, they showed reduced binding capacities, still retaining the ability to interact with T cells (even if with considerable variability between patients). In particular, the modified Ara h 2 allergen was found more efficient than the wild-type protein in reducing histamine release from RBL-2H3 cells and clinical symptoms in Ara h 2 sensitized murine models [56,57,58].

A mutant of the apple Mal d 1 allergen (a pathogenesis related protein, sharing T cell and IgE-binding epitopes with the major birch pollen allergen, Bet v 1), in which five surface-exposed amino acids were mutated, also showed a reduced allergenicity. It was demonstrated by RAST (radio allergo-sorbent test) and by SPT (skin prick test) on 14 patients with a history of birch pollinosis and apple allergy [59]. This was the first study in which a hypoallergenic mutant was evaluated on human subjects.

Five different mutants (in IgE or IgG epitopes) of the egg allergen Gal d 1 were also produced and found to have reduced capacity to bind IgG or IgE of human sera from egg allergic patients [60]. One of them was found effective in desensitization of mice previously sensitized to the third domain of Gal d 1 (one of the three trypsin-inhibitor domain of the protein [61]). They showed reduced level of histamine and anaphylaxis scores, compared to mice in which the wild-type domain was administered [62]. More recently, a Gal d 1 mutant was produced by two Cys-Ala site directed mutagenesis (involving Cys_192_ and Cys_210_) which resulted in the disruption of two separate cysteine-cysteine bridges. The mutant showed diminished IgE reactivity in immunoblot assay against a pool of egg-allergic patients’ sera [63].

The carp allergen Cyp c 1 (parvalbumin) was also expressed as mutated allergen and tested for reduced allergenicity. Mutants in the calcium binding site showed reduced IgE reactivity in immunoblot inhibition experiments performed with sera from 21 fish-allergic patients. Reduced histamine release from basophil cells was also observed. Hypoallergenicity was confirmed by SPT on a single patient and by desensitization assays in murine models [64].

Three mutants of Pru p 3, the principal allergen of peach, were produced by changing to alanine different subsets of amino acids of a single epitope. Two mutants showed reduced IgE binding with respect to the native form and did not activate basophils from allergic subjects. In addition both the mutants maintained the ability to bind IgG1 and activate T lymphocytes [65]. 

For the shrimp allergen tropomyosin (Met e 1) two mutants were constructed by site-directed mutagenesis and epitope deletion. Both molecules showed a reduced binding capacity towards IgE from eight shrimp allergic subjects and tropomyosin-sensitized mice, together with a considerable decrease in the induction of mast cell degranulation. In mice both hypoallergens induced the production of IgG antibodies able to strongly inhibit binding of Met e 1 to IgE from shrimp allergic subjects [66].

## 6. Food Allergen Databases and Web Resources

The enormous amount of data produced over the last 30 years on allergens allowed the development of several allergen related databases containing datasets and bioinformatic tools to analyse their structural features (Table 1). Accordingly, the need for a review analysis of such databases also emerged, leading to the publication of several review articles on allergen databases [32,67,68,69,70,71]. In particular, the recent review by Radauer and Breiteneder [68] gives an updated critical analysis of the available databases, including their characteristics, strengths and weakness. Readers are invited to consult these review articles to obtain detailed information on allergen databases. A few details of three databases of particular interest for food allergens are given.

### 6.1. AllergenOnline

The database was launched in 2005 as a peer-reviewed bioinformatic platform to assess the risks of suspected/candidate allergens introduced into the diet through genetically modified organisms and novel foods. Candidate proteins are identified in the literature, or in primary (NCBI) or specific databases (WHO/IUIS, Allergome) and then evaluated by a panel of experts before inclusion in the database.

### 6.2. InformAll

Basically it is a database with information on the presence of allergens in specific foods, the possibility of cross reactions with homologous allergens from other food sources, symptoms, diagnosis, etc. The database project is coordinated by the University of Manchester with the help of many European universities. The main objective of InformAll is to provide credible information by responding to the concerns and needs of various stakeholders, such as consumers, food industry, health professionals and regulators.

### 6.3. Compare

The COMPARE (COMprehensive Protein Allergen REsource) database contains an exhaustive list of clinically relevant and peer-reviewed protein allergens with citation support and species identification. It also includes descriptions of the allergens and their amino acid sequences. COMPARE was developed at HESI (Health and Environmental Sciences Institute), a non-profit institution whose mission is to collaboratively identify and help to resolve global health and environmental challenges (https://hesiglobal.org/ [86]). In particular, it is maintained by the HESI PATB (Protein Allergens, Toxins and Bioinformatics) committee, a public-private collaboration of scientists with a common interest in the assessment of allergenicity in the context of safety evaluation of novel food and feed proteins. The database also has a programmatic support from the Joint Institute for Food Safety and Nutrition (JIFSAN) at the University of Maryland (College Park, MD, USA). The latest update of the database, released on 18 January 2019 contains 2081 sequences. COMPARE is based on the application of an automated “rule-based” sorting algorithm tool, combined with a review of the literature associated with the identified sequences. The final content of the database is then established after a peer-review process conducted by a panel of allergy experts from public sectors that verify the clinical evidence of allergenicity of the newly identified sequences.

## 7. Conclusions

Ongoing progress in molecular biology, as well as in genomics, proteomics and bioinformatics, are largely contributing to the identification of food allergens and in understanding the structural basis of allergenicity. At the same time, they are also providing information and tools to improve the diagnosis and therapy of food allergies. It must be underlined, however, that for the specific aspect of immunotherapy, in vivo studies are currently restricted to animals. More studies are still required to better understand the structural and molecular aspects of allergenicity and to improve the possibility to conduct a safe immunotherapy. Other, more recently developed molecular approaches, such as epigenomics, metagenomics, and genome editing, can also contribute in developing molecular allergology. For example, an epigenomic study in peanut allergic subjects who achieved sustained unresponsiveness after a two-year period of oral immunotherapy, showed hypomethylation of CpG sites in the gene for the transcription factor FOXP3 of Treg cells [87]. The hypomethylation of the Foxp3 promoter was observed in Treg cells of mice orally sensitized to peanut proteins and subsequently subjected to epicutaneous immunotherapy (EPIT) [88]. In this study the hypermethylation of the Gata3 promoter was also detected in Th2 cells. Interestingly, mice treated with EPIT were found protected from further sensitization and maintained the acquired epigenetic signature. Recently, a review article on the epigenetics of allergies has been published [89]. The potential applications of epigenomic analysis in the diagnosis of allergies and for the evaluation of tolerance after immunotherapy (also including oral immunotherapy for food allergies) are discussed. As for metagenomics, studies conducted in children during the first year of age showed that also the intestinal microbiome may have a causal role in the development of food allergy [90,91], indicating another possible actor in the scene of food allergies. Genome editing was successfully applied to knock out the gene for the β-lactoglobulin allergen in goats, resulting in the abolishment of the protein in milk [92]. This approach can be of great help to evaluate the effects of gene modifications on the allergenicity of specific foods and to have a strong evidence of the structural relevance of specific amino acids on protein allergenicity.

## Figures and Tables

**Table 1:** List and web sites of the main databases on allergens.

Database	URL	Note	Ref.
WHO/IUIS	www.allergen.org/ [72]	a, b	[73]
SDAP	https://fermi.utmb.edu [74]	c	[75]
Allergome	http://www.allergome.org/ [76]	d	[77]
AllergenOnline	http://www.allergenonline.org/ [6]		[78]
InformAll	http://research.bmh.manchester.ac.uk/informall/allergenic-foods/ [79]		[80]
IEDB	https://www.iedb.org/ [43]		[44]
AllFam	http://www.meduniwien.ac.at/allfam/ [81]	e, f	[82]
COMPARE	https://comparedatabase.org/ [83]		--
AllerBase	http://196.1.114.46:1800/AllerBase/Home.html [84]		[85]

The databases are reported according to their year of development. (a) The database does not allow the search for food allergen; (b) the PDB entry is also reported when available; (c) last update: February 25, 2013; (d) the search for “food allergen” gives only 26 allergens; (e) last update: March 7, 2017; (f) the section dedicated to allergens having “ingestion” as route of exposition contains 66 families of allergens, with seven families (prolamin superfamily, tropomyosin, EF hand family, cupin, profiling, Bet v 1 family and thaumatin-like family) representing about 65% of the entries.
